# Comprehensive review of melatonin as a promising nutritional and nutraceutical supplement

**DOI:** 10.1016/j.heliyon.2024.e24266

**Published:** 2024-01-08

**Authors:** Waad W. Kamfar, Husam M. Khraiwesh, Mohammed O. Ibrahim, Alaa H. Qadhi, Wedad F. Azhar, Khloud J. Ghafouri, Maha H. Alhussain, Abdullah F. Aldairi, Abdullah M. AlShahrani, Abdullah F. Alghannam, Rwaa H. Abdulal, Abed H. Al-Slaihat, Maysoun S. Qutob, Mahmoud E. Elrggal, Mazen M. Ghaith, Firas S. Azzeh

**Affiliations:** a Department of Clinical Nutrition, Faculty of Applied Medical Sciences, UmmAl-Qura University, P.O. Box: 7067, Makkah, Saudi Arabia; b Medical Nutrition and Food Services Department, Almana Hospitals, Aziziah, Dammam, Saudi Arabia; c Department of Nutrition and Food Technology, Faculty of Agriculture, Mu'tah University, Karak, Jordan; d Department of Food Science and Nutrition, College of Food and Agriculture Sciences, King Saud University, Riyadh, 11451, Saudi Arabia; e Faculty of Applied Medical Sciences, Department of Clinical Laboratory Sciences, Umm Al-Qura University, Al Abdeyah, Makkah, 7607, Saudi Arabia; f Department of Basic Medical Sciences, College of Applied Medical Sciences, Khamis Mushayt, King Khalid University, Abha, 62561, Saudi Arabia; g Lifestyle and Health Research Center, Health Sciences Research Center, Princess Nourah Bint Abdulrahman University, Riyadh, 84428, Saudi Arabia; h Vaccines and Immunotherapy Unit, King Fahd Medical Research Center, King Abdulaziz University, Jeddah, 21589, Saudi Arabia; i Department of Biology, Faculty of Science, King Abdulaziz University, Jeddah, 21589, Saudi Arabia; j Department of Nutrition and Food Technology, School of Agriculture, The University of Jordan, Amman, Jordan; k Clinical Nutrition and Dietetics Department, Faculty of Allied Medical Sciences, Applied Science Private University, Amman, Jordan; l College of Pharmacy, Umm Al-Qura University, Makkah, Saudi Arabia; m Department of Nutrition and Food Processing, Faculty of Agricultural Technology, Al-Balqa’ Applied University, Salt, Jordan

**Keywords:** Anti-inflammatory agent, Antioxidant, Melatonin, Melatonin receptors

## Abstract

**Background:**

Melatonin is an indoleamine hormone secreted by the pineal gland at night and has an essential role in regulating human circadian rhythms (the internal 24-h clock) and sleep-wake patterns. However, it has recently gained considerable attention for its demonstrated ability in disease management. This review discusses the major biological activities of melatonin, its metabolites as nutritional supplements, and its bioavailability in food sources.

**Methods:**

The information acquisition process involved conducting a comprehensive search across academic databases including PubMed, Scopus, Wiley, Embase, and Springer using relevant keywords. Only the most recent, peer-reviewed articles published in the English language were considered for inclusion.

**Results:**

The molecular mechanisms by which melatonin induces its therapeutic effects have been the subject of various studies.

**Conclusion:**

While melatonin was initially understood to only regulate circadian rhythms, recent studies indicate that it has a far-reaching effect on various organs and physiological systems, such as immunity, cardiovascular function, antioxidant defense, and lipid hemostasis. As a potent antioxidant, anti-cancer, anti-inflammatory, and immunoregulatory agent, multiple therapeutic applications have been proposed for melatonin.

## 1 Introduction

Over the past few years, maintaining optimal health has become increasingly dependent on a healthy lifestyle, including sleep and diet. Consequently, there has been an increase in demand for foods that have beneficial effects on the human body, leading to the rapid development of new food products, known as nutraceuticals [1].

Melatonin (MLT), also known as N-acetyl-5-methoxytryptamine, is a type of indoleamine derived primarily from the essential amino acid tryptophan (TRP), which is produced by the pineal gland and directly released into the bloodstream of vertebrates [2]. It is also synthesized in several other organs, including the heart, gastrointestinal tract, skin, bone marrow, and lymphocytes [3,4]. Lerner and colleagues first isolated this molecule from a cow pineal gland in 1958 [5].

MLT is an essential component in the regulation of circadian rhythm in humans, specifically the sleep cycle [6]. Several studies initially demonstrated that MLT has a sleep-regulating effect due to its production during the night [2,7]; it is therefore widely used as a supplement for the treatment of sleep disorders such as insomnia, anxiety, and jet lag [3]. Several forms of MLT are available over-the-counter, including capsules, tablets, and liquids that contain a complex mixture of vitamins and minerals [8,9].

Numerous diseases, including cardiovascular diseases (CVDs), coronavirus disease (COVID-19), obesity, and cancer, have been linked to insufficient MLT secretion [[10], [11], [12]]. MLT has been shown to exhibit strong antioxidant properties and is capable of protecting against oxidative stress [2]. These antioxidant capabilities enable MLT to scavenge free oxygen radicals and prevent oxidative damage to cells and tissues; much research has therefore focused on the therapeutic and protective role of MLT in regulating human hemostasis through its anti-inflammatory, antioxidant, anti-infective, and anti-tumor properties [13].

This review summarizes recent discoveries relating to the efficacy of MLT on health, investigates its biosynthesis and metabolism, discusses potential mechanisms of action, and outlines the dietary sources of MLT-rich foods and supplemental guidance.

## 2 Pineal gland and the melatonin circadian rhythm

### 2.1 Synthesis, secretion, and metabolism of melatonin

Physiologically, MLT levels rise from birth and decline with age. MLT is synthesized 24 h a day; however, it is produced and released into the bloodstream more at night [9]. Its secretion rises during the night and peaks between 2 a.m. and 4 a.m., before gradually declining to its base level for the rest of the day [14]. Darkness increases its synthesis and secretion, while light inhibits it [15], with the retina transmitting relevant information to the pineal gland via the suprachiasmatic nucleus of the hypothalamus [3,16]. Its serum concentration varies between 80 and 120 pg/mL at night, gradually decreasing to 10–20 pg/mL during the day [3].

MLT is not stored in the pineal gland but is released into the bloodstream, where it is rapidly degraded by the liver [17]. It is primarily metabolized by cytochrome P450 in the liver [18]; its major metabolite, 6-sulfatoxymelatonin (aMT6s), is produced in the liver and excreted in the urine [19]. CYP1A1 and CYP2C19 metabolize MLT into 6-hydroxymelatonin (6-OM) and N-acetylserotonin (NAS), which are mainly converted to conjugated sulfates by sulfotransferases in the liver and excreted in the urine. CYPA2B or 2,3-indoleamine dioxygenase degrades a small amount of MLT in tissue such as the brain and skin, producing 6-OM or N-Acetyl-N-formyl-5-methoxykynurenamine (AFMK). AFMK is not thought to be a major metabolic route, due to its water solubility [18].

MLT may be bound to albumin and hemoglobin as part of the circulatory system, but is primarily transported by the serum albumin [17]. Its amphiphilic nature allows MLT to cross both cellular and morphophysiological barriers, including the blood-brain barrier (BBB) [3]. Due to its lipophilic and hydrophilic properties, it is permeable to cell membranes and can be found in other body fluids, including saliva, urine, milk, sperm, and amniotic fluid [14]. It has been reported that the consumption of foods containing MLT may increase serum levels of MLT and urine levels of aMT6s [3].

**Fig. 1:**
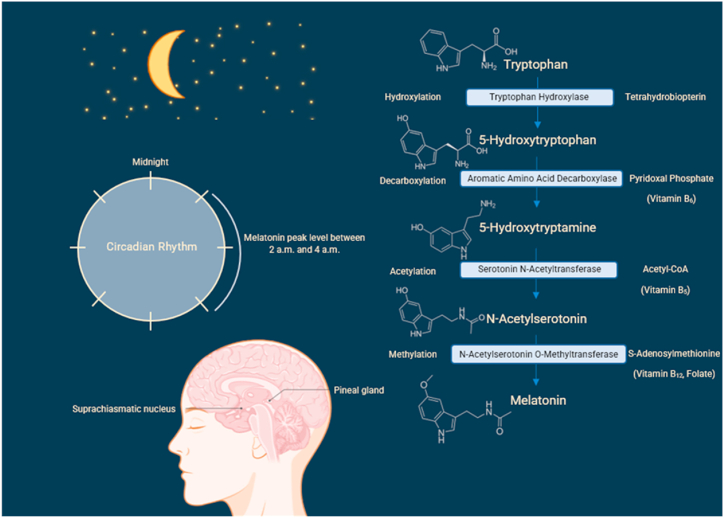
Melatonin synthesis and secretion in the pineal gland (Note: This figure is self-designed by the authors using Biorender® software).

TRP, an amino acid found in food, is required for the biosynthesis of MLT. Vitamins B6, B5, B12, and folate act as co-factors in the conversion of L-5-hydroxytryptophan to 5-hydroxytryptamine (serotonin), which is required for MLT synthesis [3]. The retina, gastrointestinal tract, skin, lymphocytes, and bone marrow can also produce MLT, in addition to the pineal gland [17]. The synthesis of MLT involves six enzymes and begins with the hydroxylation of TRP by tryptophan-5-hydroxylase to produce L-5-hydroxytryptophan. This is then decarboxylated to serotonin by L-aromatic amino acid decarboxylase, and NAS is then produced by arylalkylamine N-acetyltransferase. As a final step, NAS is converted into N-acetyl-5-methoxytryptamine (MLT) by N-acetylserotonin O-methyltransferase [2,20]. MLT is therefore synthesized by four biochemical reactions: hydroxylation, decarboxylation, acetylation, and methylation [2]. These reactions occur in the pinealocytes, and the light/dark cycle then regulates MLT secretion [21], as illustrated in Fig. 1.

## 3 Melatonin receptors and their mechanisms of action

### 3.1 Melatonin receptor subfamily

In humans, there is an MLT receptor subfamily consisting of MT1 and MT2, which bind MLT as their natural ligand, and GPR50 [19]. The retinoid Z receptor/retinoic acid receptor-related orphan receptor (RZR/RORα) is the fourth MLT receptor type [20]. The transmembrane spanning proteins MT1 and MT2 belong to the G-protein coupled receptor superfamily, which exhibits high-affinity binding and is activated by low concentrations of MLT [22,23]. The MT1 receptor consists of 350 amino acids coupled to Gi and Gq/11 and is located in the brain, cardiovascular system, immune system, testes, ovary, skin, liver, kidney, adrenal cortex, placenta, breast, retina, pancreas, and spleen. The brain's MT1 receptors are primarily found in the hypothalamus, cerebellum, substantia nigra, and ventral tegmental area [23]. Meanwhile, the MT2 receptor has 363 amino acids, which are 70 % similar in distribution to the MT1 receptor; it is also associated with a G-protein and participates in activating Gi. GPR50 consists of 617 amino acids, a relatively long C-tail, and a high sequence homology (50 %) with MT1 and MT2 [22]. This receptor (an MLT-related orphan receptor) does not bind to MLT but enhances the binding of MLT to the MT1 receptor [10]; moreover, MLT functions through this receptor to regulate the retinoic acid receptor superfamily transcription factors [17].

In contrast, MT3 is a cytoplasmic receptor located in the cytoplasm of many tissues, including the liver, lung, kidney, eye, heart, brown fat, intestines, and muscles [24]. Furthermore, it is a quinone reductase 2 enzyme, which inhibits the electron transfer reactions of quinones, preventing oxidative stress, which is a significant part of the body's protection against free radicals [25].

### 3.2 Binding to melatonin receptors in plasma membrane, cytoplasm, and nucleolus

Most of MLT's actions are mediated by its interactions with the membrane-bound G-protein coupled MLT receptors MT1 and MT2, the quinone reductase II enzyme (MT3), or indirectly by RZR/RORα [3]. Different types of cells contain specific receptors and intracellular targets for MLT that control a variety of physiological processes, including the activity of adenylate cyclase, guanylate cyclase, phospholipase C (PLC), calcium, and potassium channels in the cell membrane [16].

The activation of MT1 or MT2 by MLT leads to a decrease in cyclic adenosine monophosphate (cAMP) and, therefore, the inhibition of protein kinase A. The MT1 receptor inhibits adenylate cyclase by activating the Gi subunits, thus reducing cAMP production. MT2 receptors act to inhibit the formation of cyclic guanosine monophosphate (cGMP) [8]. In addition, MT2 interferes with cGMP formation by inhibiting guanylyl cyclase. Furthermore, the Gq subunit of MLT induces PLC activity and intracellular calcium concentrations [26].

In smooth muscles, MLT is able to bind to calcium-binding proteins (such as calmodulin) with high affinity and inhibits the activation of myosin light chain kinase, decreasing contractability [14]. MLT can interact with molecular effectors, including calcium/calmodulin-dependent kinase II, and directly scavenge free radicals. MT3 may impact the expression of antioxidant and detoxification enzymes and reduce proliferation. Researchers have reported that membrane MLT receptors are present in almost all types of cells, including the retina, brain, suprachiasmatic nucleus, pituitary gland, ovary, cerebral and peripheral arteries, kidney, pancreas, fat, and immune cells [22,27], resulting in physiological functions such as reproduction, cardiovascular regulation, and immune function [3,28]. MLT also modulates the activity of RZR/RORα, regulating the expression of their target genes. Following MLT stimulation, RZR/RORα has been demonstrated to bind DNA, thus increasing the transcription of mRNA coding for γ-glutamylcysteine synthetase, which is the limiting enzyme in glutathione synthesis. As a result, the enzyme and glutathione are expressed to protect the cell from oxidative stress and regulate the cell cycle [29]. MLT's well-known antioxidant activity is partly due to its direct interaction with reactive oxygen and nitrogen species and its ability to activate antioxidant receptors [30].

### 3.3 Antioxidant effect

**Fig. 2:**
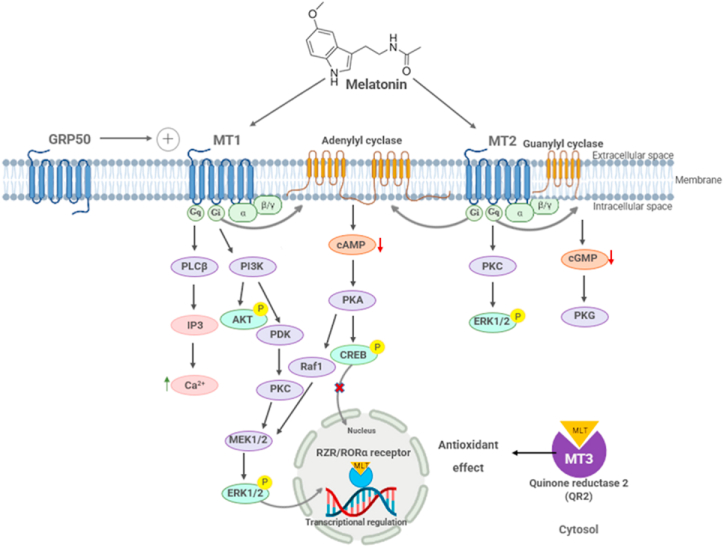
Melatonin receptor signaling pathways of antioxidant mechanisms.

Oxidative stress is a condition in which the equilibrium between the production of reactive oxygen species (ROS) and the body's ability to eliminate them is disturbed [18]. MLT can be considered one of the most potent antioxidants in nature. Unlike some traditional antioxidants, such as vitamins C and E, MLT binds up to ten free radicals per molecule [31], acting on reactive oxygen and nitrogen species and the cellular antioxidant enzyme system [22]. Two main mechanisms explain MLT's antioxidant and free radical scavenging properties, as illustrated in Fig. 2. The first mechanism involves MLT binding to the MT3 receptor, which controls the electron transfer reactions of quinones and prevents oxidative stress. The second mechanism involves MLT entering the nucleus of the brain and binding to transcription factors RZR/RORα [32]. Interaction with RZR/RORα is essential for immune modulation and antioxidant enzyme regulation [33]. In addition to the two mechanisms mentioned above, MLT also indirectly stimulates antioxidative enzymes such as glutathione peroxidase, glutathione reductase, superoxide dismutase, and glucose-6-phosphate dehydrogenase, which prevents cellular damage under conditions of excess oxidative stress [32]. In addition to preserving mitochondrial function, MLT also inhibits subsequent apoptosis and cell death by reducing mitochondrial oxidative stress [34]. Since MLT is highly lipophilic, it can easily cross the cell membrane and reach intracellular compartments, including nuclei and mitochondria. Antioxidative enzymes are stimulated by activating the MT1 and MT2 receptors [10]; the upregulation of these receptors leads to the antioxidative defense systems being upregulated, which increases the activity of antioxidant enzymes such as superoxide dismutase and glutathione peroxidase [3]. Additionally, MLT exerts neuroimmunomodulatory effects on the immune system via its membrane receptors, which have been found in immune cells, tissues, bone marrow mononuclear cells, leukocytes, and even subcellular compartments [35]. MLT has been found to inhibit the production of forskolin-stimulated cAMP, cGMP, and diacylglycerol, which results in improved immunity [18].

The actions of MLT are mediated by interactions with the G-protein-coupled membrane-bound MLT receptors MT1 and MT2, the quinone reductase II enzyme (MT3), and by indirect interactions with the nuclear orphan receptors of the RZR/RORα family. In response to the MLT activation of MT1 receptors, Gα_i_ is activated, resulting in a decrease in levels of the secondary messenger cAMP and Gβγ‐dependent activate PI3K/Akt, PKC, and extracellular signal-regulated kinase (ERK) pathways. The coupling of MT1 to G_q_ results in the activation of PLC and an increase in intracellular Ca^2+^. Moreover, in response to the MLT activation of MT2 receptors, G_αi_‐dependent is activated, resulting in a decrease in the cAMP and ERK signaling pathways and inhibiting cGMP levels. cAMP, cyclic adenosine monophosphate; cGMP, cyclic guanosine monophosphate; IP3, inositol triphosphate; MT1-MT3, melatonin specific receptor 1, 2, 3; PLC, phospholipase C; QR-2, quinone reductase 2; ROS, reactive oxygen species; RZR/RORα, retinoid Z receptor/retinoic acid receptor-related orphan receptor.

## 4 Nutrition and melatonin

### 4.1 Dietary sources of melatonin

The consumption of MLT-rich foods is associated with health benefits, contributing to higher serum concentrations and antioxidant levels in humans, and these foods are therefore considered to be nutraceuticals [35]. MLT is synthesized in the enterochromaffin cells of the gastrointestinal tract, and there is at least 400 times more MLT in the gastrointestinal tract than in the pineal gland [36]. The release of this MLT into the circulation is correlated with the frequency of the foods eaten, particularly TRP consumption; the concentration of MLT in the gastrointestinal tract exceeds blood levels by 10–100 times. A study found that MLT-rich foods are positively correlated with clinical-metabolic markers [3].

In recent decades, MLT has been detected in animal foods and edible plants due to its powerful effects on health, with concentrations varying markedly among different foods, as presented in Table 1; levels can also change during food preparation [3]. The content of MLT in food has also been shown to vary widely between species [35], with considerable differences in MLT concentrations being reported for a variety of food species and organs, ranging from pg/g to mg/g [31]. It has also been established that MLT-rich foods may influence human health via an increase in MLT serum levels and antioxidant capacity [18]. MLT can be distributed unevenly among individual animals and plants due to the different biophysical dynamic features of individual organs [31].

**Table 1:** An analysis of melatonin content in selected foods.

Food	Scientific Name	Method of Analysis	Melatonin content	Reference
Fruits				
Pineapple	Ananas comosus	GC/MS	0.28 ng g−1	[37]
Strawberry	Fragaria magna	GC/MS	0.14 ng g−1	[38]
Banana	Musa ensete	GC/MS	0.66 ng g−1	[39]
Apple	Malus domestica	GC/MS	0.16 ng g−1	[39]
Pomegranate	Punica granatum	GC/MS	0.17 ng g−1	[39]
Grape	Vitis vinifera Merlot	HPLC-MS/MS	3.9 ± 0.1 ng g−1	[39]
Kiwi fruit	Actinidia chinensis L.	HPLC-FD	0.02 ng/g	[35]
Cranberry	Vaccinium oxycoccos L.	UPLC-MS	40 ± 10 μg/g DW	[35]
Grape	Albana, white	HPLC-FD	1.2 ng/g	[38]
**Vegetables**				
Onion	Allium cepa L.	GC/MS	0.30 ng/g FW	[37]
Garlic	Allium sativum L.	GC/MS	0.59 ng/g FW	[37]
Cabbage	Brassica oleraceae L.	GC/MS	0.31 ng/g FW	[37]
Cauliflower	Brassica oleraceae L.	GC/MS	0.82 ng/g FW	[37]
Turnip	Brassica rapa L.	GC/MS	0.50 ng/g FW	[37]
Broccoli	Brassica oleraceae L.	ELISA	0.41 ± 0.04 ng/g DW	[35]
Mushroom	Agaricus bisporus	RP-HPLC	4300–6400 ng/g DW	[35]
Cucumber	Cucumis sativus L.	GC/MS	0.59 ng/g FW	[37]
Carrot	Daucus carota L.	GC/MS	0.49 ng/g FW	[37]
Radish	Raphnus sativus L.	GC/MS	0.76 ng/g FW	[37]
Ginger	Zingiber officinale Rosc	GC/MS	1.42 ng/g FW	[37]
Black olive	Not specified	LC-MS/MS	0.01 ng/g DW	[35]
Beetroot	Beta vulgaris	GC/MS	0.002 ng/g	[35]
Purslane	Portulaca oleracea L.	GC/MS	19 ng/g WW	[35]
Spinach	Not specified	GC/MS	0.04 ng/g WW	[35]
Asparagus	Asparagus officinalis L.	HPLC-FD	0.01 ng/g FW	[35]
Pepper	Capsicum annuum L.	UHPLC-MS/MS	4.48 ng/g FW/31.01 ng/g DW	[35]
Tomato	Lycopersicon esculentum L.	HPLC-FD	0.03 ng/g FW	[38]
Corn	Not specified	GC/MS	1.88 ng/g FW	[37]
**Cereals**				
Oat	Avena sativa L.	HPLC-FD	1.80 ng/g FW	[3]
Rice	Oryza sativum L.	HPLC-FD	1.01 ng/g FW	[3]
Wheat	Triticum aestivum L.	HPLC-ECD	124.7 ± 14.9 ng/g FW	[18]
Barley	Hordeum vulgare L.	GC/MS	0.87 ng/g FW	[37]
**Legumes**				
Lentils	Lens culinaris L.	HPLC-MS/MS	0.5 ng/g DW	[35]
Kidney beans	Phaseolus vulgaris L.	HPLC-MS/MS	1.0 ng/g DW	[35]
Soybean	Glycine max	RIA	0.45 ± 0.03 ng/g DW	[35]
**Nuts**				
Pistachio	Pistacia vera L.	GC/MS	233,000 ng/g DW	[18]
Walnuts	Juglans regia L.	HPLC–MS	1.02 ± 0.06 ng/g FW	[35]
**Animal Foods**				
Lamb	Not specified	HPLC	1.6 ± 0.14 ng/g	[31]
Beef	Not specified	HPLC	2.1 ± 0.13 ng/g	[31]
Salmon	Not specified	HPLC	3.7 ± 0.21 ng/g	[31]
Chicken	Not specified	HPLC	2.3 ± 0.23 ng/g	[31]
Egg	Not specified	HPLC	6.1 ± 0.95 ng/g	[31]
Yoghurt	Not specified	LC-MS/MS	0.13 ± 0.01 ng/mL	[35]
Cow milk	Not specified	LC-MS/MS	14.45 ± 0.12 pg/mL	[35]
**Human milk**				
Breast milk	Not specified	ELISA	0–42 pg/mL	[31]

#### 4.1.1 Animal foods

Tan and his colleagues reported, for the first time, that MLT can be found in certain types of meat, including chicken, lamb, beef, and fish [31]. TRP is an essential amino acid for humans. Since humans cannot produce TRP sufficiently, this amino acid must be absorbed from protein-containing foods through the small intestine. MLT is produced by metabolizing TRP via the serotonin pathway [40], and the BBB must be crossed in order to transport TRP into the brain for MLT synthesis. Then TRP competes with isoleucine, leucine, phenylalanine, tyrosine, and valine, which are large neutral amino acids, and a higher plasma TRP/LNAA ratio (mol/mol) is therefore desirable for TRP transport through the BBB. Researchers have used plasma TRP/LNAA ratios in animal and human studies to measure serotonin synthesis in the brain. The contents of a meal influence this ratio, with TRP-rich proteins increasing it [40,41]. It appears that a diet rich in meat, poultry, fish, eggs, and milk may contribute to MLT levels, probably due to the high content of TRP and vitamin B_12_, which is able to increase the amount of TRP in the brain and the number of MLT receptors in the brain [6]. MLT levels are higher in eggs and fish compared to meat; in particular, MLT levels in solid dried eggs are 6.1 ± 0.95 ng/g, whereas those in salmon are 3.7 ± 0.21 ng/g [31].

#### 4.1.2 Human milk

Reppert and colleagues (1993) studied MLT for its potential physiological role in human milk, demonstrating that it could be transmitted into neonates through human milk by measuring human milk [39]. The gastrointestinal tract of newborns and infant mammals contains MLT from the mother, since it transmits efficiently across the placenta and is absorbed from the mother's milk [29]. MLT concentrations follow a circadian pattern in preterm and term breast milk, with peaks occurring at night and declining during the day [42]. According to a study by Mayo and co-workers, high concentrations of MLT may stimulate the production of antioxidant enzymes in breast milk. There was no correlation between gestational age and MLT concentration. It is noteworthy that bottled milk formulations do not contain MLT [39].

Human colostrum contains immune-competent cells (colostral mononuclear cells), capable of synthesizing MLT in an autocrine manner during the first 4 or 5 days after birth [43]. Based on the observation that MLT concentrations in milk were approximately ten times greater at night than in the daytime, night milking may enhance the health benefits of breast milk [42]. The high level of TRP present in human milk can also be converted into MLT [42]. Furthermore, the concentration of MLT in colostrum was found to be similar to plasma [44], which could benefit newborn infants whose rhythmic excretion of MLT is deficient in the early weeks of life [39]. Artificial formulas and fermented milk drinks did not contain detectable MLT levels [45].

#### 4.1.3 Plant foods

MLT was initially reported in edible plants in 1995 [18]. TRP acts as a precursor to both MLT and indole-3-acetic acid, suggesting that both have similar physiological roles [46]. Recent studies suggest that it may also have analogous functions in plants, including controlling the circadian rhythm and exhibiting potent antioxidant properties [46,47]. Also, some plant species exhibit circadian and circannual variations in MLT levels. Before determining the effects of a particular diet, it is necessary to identify the whole composition of the food [37]. The presence of MLT has been identified in various components of vegetable origin in the human diet, including plants, legumes, fruits, nuts, and products derived from plants [31]. Plant foods containing MLT are an essential part of the diet and provide significant amounts of the MLT required by the body. Plant foods containing MLT are an essential part of the diet and provide significant amounts of the MLT required by the body; research indicates that their consumption can increase levels of MLT and antioxidants in the blood [18]. An essential characteristic of seeds and other reproductive organs is their high lipid content and vulnerability to oxidative stress [3].

MLT has been found in many commonly consumed fruits. The fruits most commonly studied for their MLT content are grapes, cherries, and strawberries, demonstrating differences in their cultivars [35]. MLT has also been detected in many plant-derived foods, including nuts, fruits, seeds, cereals, oils, and coffee beans [31]. Many vegetables contain MLT, but the level is undetectable in potatoes and low in beets. The most studied vegetables are tomatoes and peppers, which contain relatively high levels of MLT within the vegetable group [31]. Recently, MLT also was found in a variety of legumes, which are essential sources of protein, vitamins, and minerals. Recent research has focused on the benefits of these foods with regard to disease prevention, due to their fiber content, slow digestion of starch, prebiotic oligosaccharides, and phenolic content [39]. Some of these compounds also possess antioxidant properties that may contribute to their potential health benefits in preventing diseases caused by oxidative stress [39].

Some human experiments have been conducted with fruits, demonstrating that the consumption of sweet cherries, plums, or grape juice by young, middle-aged, and elderly subjects increased urinary levels of aMT6s [31]. A crossover study showed that serum MLT levels increased up to fivefold after consumption of tropical fruits, such as bananas, oranges, and pineapple [18]. Moreover, the level of MLT was significantly higher in nuts and medicinal herbs [3].

### 4.2 Effects of melatonin rich foods on circulating levels

Recent studies concluded that the plasma level of MLT is influenced by dietary intake [39]. The endogenous secretion of MLT decreases after childhood, and it is possible to increase the dietary intake of this hormone. Evidence reveals that the consumption of foods rich in MLT may have health benefits linked to increasing circulating MLT levels [48]. Published studies have investigated either plasma MLT levels or the excretion of the urine metabolite of MLT, known as aMT6s [39]. The oral administration of MLT results in rapid absorption, wide distribution, and complete metabolism. It is capable of passing through the BBB [49]. There were significant increases in circulating MLT levels in humans after consuming food containing MLT [35]. Glucose-rich food sources enhance the passage of TRP through the BBB [49]. One indirect method of determining the efficacy of MLT-rich foods in raising MLT circulation is to monitor the excretion of aMT6s, the primary metabolite of MLT in humans, resulting from hydroxylation and sulfation of MLT in the liver [50]. According to a study published in 1969, 60 % of injected MLT appears as aMT6s [39].

All values are presented as the mean ± SD. Data were collected from all studies from 2017 to 2022. Abbreviation used in the Table: ELISA: enzyme-linked immunosorbent assay; LC: liquid chromatography; MS: mass spectrometry; GC: gas chromatography/mass spectrum; HPLC: high-performance liquid chromatography; RIA: radioimmunoassay; UHPLC: ultra-high-performance liquid chromatography; ECD: electron capture detector; FD: fluorescence detector; RP-HPLC: reversed-phase high-performance liquid chromatography; DW: dry weight; FW: fresh weight.

## 5 Melatonin as a nutraceutical supplement

### 5.1 Introduction

The term nutraceutical was coined in 1989 by Stephen De Felice, founder and chairman of the Foundation for Innovation in Medicine, an organization in the United States that promotes medical health research. According to him, nutraceuticals are foods or parts of foods beneficial to human health, including the prevention and treatment of disease [51]. Another definition of nutraceutical is a combination of two terms, nutrition/nutrients (nutritional products) and pharmaceuticals (medicines or drugs), found in foods and food component products containing active principles from plants that provide health and medical benefits, including the prevention and treatment of diseases [51]. Initially, several studies demonstrated that exogenous MLT effectively improved sleep, most likely because of its chronobiotic properties, and it has been shown to contribute to adjusting and maintaining a regular circadian rhythm [3]. However, there are many other health effects associated with the application of MLT: MLT supplementation, for example, is beneficial for inflammation markers, hypertension, oxidative stress, and metabolic syndrome [3]. In addition to being available over-the-counter, MLT can also be obtained via medical prescription [52]. In contrast, drugs used to treat conditions such as chronic inflammation may have undesirable side effects, while nutraceuticals such as MLT are known to be beneficial in treating inflammation due to their high safety and tolerability [53].

### 5.2 Bioavailability of exogenous melatonin

The oral administration of MLT results in rapid metabolism within approximately 30–50 min, usually to 6-OM and NAS in the liver. Almost 80 % of administered MLT is excreted in urine as its sulfate conjugates [39]. There are two pathways through which MLT is catabolized: enzymatic and nonenzymatic [54]. In humans, the primary enzyme responsible for liver catabolism is cytochrome P450, leading to the release of 6-OM, where CYP1A2 is the predominant enzyme (Nelson et al., 2004). CYP2C9 and CYP2C19 are two other CYP450s involved to a lesser extent in the enzymatic degradation of MLT into 6-OM. Conjugation of 6-OM with sulfuric acid or glucuronic acid, followed by excretion in urine, significantly reduces the bioavailability of the exogenous dose. Free radicals, such as hydroxyl radicals, are involved in the nonenzymatic 6-hydroxylation process of MLT, representing a minor catabolic pathway. MLT has a bioavailability of 9–33 % for oral administration in humans, with a time to peak concentration (T_max_) of approximately 50 min following the administration of oral immediate-release formulations. Formulations promoting the release of MLT in selected sections of the gastrointestinal tract, such as the ileum, where the greatest absorption of MLT occurs, would increase MLT absorption, but it is unclear whether this translates into improved bioavailability, considering that MLT is metabolized into 6-OM by CYP enzymes [54]. Exogenous MLT is capable of diminishing oxidative stress by inhibiting the formation of hydroxyl radicals and inhibiting the proliferation, inflammation, and apoptosis of cells. MLT can indirectly enhance the function of antioxidant enzymes such as glutathione, glutathione peroxidase, and superoxide dismutase. MLT also inhibits nuclear factor-kB translocation and pro-inflammatory mediator production [53]. According to previous studies, serum concentrations of C-reactive protein (CRP), interleukin 6 (IL-6), and MLT were affected by age, dosage, and treatment duration [55]. A study conducted by Zarezadeh et al. (2020) indicated that the administration of MLT for ≥12 weeks at a dosage of ≥10 mg/day is more efficacious at amending IL-6 and tumor necrosis factor-alpha (TNF-α) levels, suggesting that long-term interventions with high doses of MLT are required to effectively reduce inflammation.

### 5.3 Guidance on regulating dietary supplements of melatonin

MLT is widely used throughout the world as a food supplement to counteract physiologically diminished pineal gland activity, including in the United States and Europe [17]. It is regulated differently by the American Food and Drug (FDA) Administration and the European Food Safety Authority (EFSA). According to the EFSA, MLT is only acceptable for sleeping disorders and jet lag [56]. Some European countries have restricted MLT to decreasing quantities [12]. However, MLT has been acknowledged as a safe food supplement that does not cause severe adverse reactions [3]. To date, only a few studies have investigated exogenous MLT administration and its side effects with possible adverse reactions as their primary objective. Anderson and colleagues summarized the safety of MLT administration in humans, in both infants and adults. As reported throughout the literature, the most common side effects include dizziness, headache, nausea, and sleepiness [57].

### 5.4 Melatonin analogues

MLT is commercially available worldwide in numerous dosage forms and formulations, ranging from 1.99 mg to 20 mg [58]. Since MLT has a relatively short half-life (0.57–0.67 h), the need for longer-acting analogues has led to the creation of compounds such as ramelteon, agomelatine, and tasimelteon. Ramelteon has a half-life of 1.5–2 h, while its metabolite has a longer half-life of 2–5 h. Ramelteon has a higher affinity for MLT receptors (3–16 times that of MLT) and is recommended in doses of 8 mg to treat insomnia, decrease sleep latency, and increase sleep duration. The drug was previously known as TAK-375 and was commercially marketed as Rozerem®. Agomelatine and tasimelteon reach peak levels after 1–2 h and 0.5–3 h, respectively. Agomelatine is indicated for treating sleep disorders caused by major depressive disorders and was previously known as S20098, commercially marketed as Valdoxan®. The dosage of this medication is 25 mg and it is well absorbed by the body (80 %). Tasimelteon is indicated for non-24 h sleep-wake disorders when administered at 20 mg. The drug was previously known as BMS-214778 and was commercially marketed as Hetlioz® [24].

The doses used in studies have ranged from 0.1 to 10 mg [59]. There has been limited research conducted, and no evidence has been provided regarding the safety or effectiveness of MLT beyond 16 weeks of administration [60].

### 5.5 Phytomelatonin (PHT-MLT) vs. synthetic melatonin (SNT-MLT)

Phytomelatonin (PHT-MLT) is the term used for MLT of plant origin [61]. Blask and colleagues discovered this term in edible plants, named PHT-MLT in 2004 [62]*.* PHT-MLT has been found in various vegetables, seeds, fruits, nuts, and aromatic/medicinal herbs [31,63]. Synthetic melatonin (SNT-MLT) is used to produce commercial MLT products [38]. The potential use of PHT-MLT in dietary supplements stems from the desire to avoid the ingestion of a variety of by-products produced during the chemical process of MLT synthesis. By-products such as 1,2,3,4-tetrahydro-β-carboline-3-carboxylic acid, 3-(phenylamino)-alanine, and 1,1′-ethylidenebis-(tryptophan) are some of the most common synthetic compounds that can appear in SNT-MLT preparations, listed in Table 2, and are generally tryptophan-related compounds. These include 1,1′-ethylidenebis-(l-tryptophan), called peak E, which is related to eosinophilia-myalgia syndrome, a chronic multi-systemic disease characterized by eosinophilia and subacute myalgia, due to defective criteria for the manufacture of TRP [47,64]. The disease eosinophilia-myalgia syndrome was first discovered in October 1989 in three patients from the United States who were all taking L-TRP dietary supplements [64]. The risk of ingesting these contaminants is much lower for MLT supplements since the recommended daily dose is 1000 times lower than L-TRP supplements [63].

**Table 2:** Some of the most common synthetic compounds which can appear in synthetic melatonin preparations.

1,2,3,4-tetrahydro-β-carboline-3-carboxylic acid
3-(phenylamino)-alanine
1,1′-ethylidenebis-(l-tryptophan)
2-(3-indolylmethyl)-tryptophan
Formaldehyde melatonin
Formaldehyde-melatonin condensation products
Hydroxymelatonin isomers
5-hydroxy-tryptamine derivatives
5-methoxy-tryptamine derivatives
N-acetyl- and diacetyl- indole derivatives
Hydroxy-bromo-propylphthalimide
Chloro-propylphthalimide
1,3-diphthalimidopropane

According to Ref. [65], dietary supplements containing PHT-MLT are marketed in New Zealand, Spain, and the United States. Perez-Llamas and his co-workers describe the three PHT-MLT products as containing an extract of a single component: freeze-dried Montmorency tart cherry skin (*Prunus cerasus*). The Spanish and US products contain a complex mixture of ingredients extracted from dried plants and herbs with a high MLT content that the Spanish manufacturer has not disclosed. Specifically, these products are made from alfalfa (*Medicago sativa*), chlorella powder (*Chlorella Vulgaris*), and rice (*Oryza sativa*). Another PHT-MLT product of Chinese origin contains an extract of St. John's Wort [65]. A recent study by Ref. [61] demonstrated that a PHT-MLT extract containing plant-based MLT in a herbal complex of three components could reduce intracellular free radical levels in laboratory models under a wide range of *in vitro* assay circumstances. Comparing PHT-MLT to SNT-MLT, the antioxidant properties improved the intestinal absorption of MLT from natural sources, making PHT-MLT more effective with regard to antioxidant properties and the cyclooxygenase 2 (COX-2) inhibitory properties from the SNT-MLT solution [61].

## 6 Conclusion

MLT is a ubiquitous molecule in human body organs, including the pineal gland, heart, bone marrow, and lymphocytes. This molecule has been generating significant interest in the past decade, which is available as over-the-counter supplements and perceived as a natural and safe product. Currently, MLT is commercially available either synthetic or as PHT-MLT products. However, PHT-MLT is a MLT of plant extracts. The health benefits derived from the dietary intake of MLT are until now controversial, as it is not recognized if the chronic consumption of melatonin through the diet has physiological effects. In contrast, SNT-MLT preparations include unwanted by-products. Thus, using PHT-MLT instead of SNT-MLT in many applications such as dietary supplements and pharmaceutical products ensures that PHT-MLT-rich products are free of pesticides and other contaminants. Using PHT-MLT instead of SNT-MLT will ensure no unwanted by-products in MLT treatments.

## Data availability

No data was used for the research described in the article.

## Additional information

No additional information is available for this paper.

## CRediT authorship contribution statement

**Waad W. Kamfar:** Conceptualization, Data curation, Investigation, Methodology, Writing – original draft, Writing – review & editing. **Husam M. Khraiwesh:** Software, Validation, Visualization, Writing – original draft, Writing – review & editing. **Mohammed O. Ibrahim:** Validation, Visualization, Writing – review & editing. **Alaa H. Qadhi:** Funding acquisition, Resources, Writing – original draft, Writing – review & editing. **Wedad F. Azhar:** Supervision, Writing – original draft, Writing – review & editing. **Khloud J. Ghafouri:** Investigation, Resources, Visualization, Writing – original draft, Writing – review & editing. **Maha H. Alhussain:** Data curation, Writing – original draft, Writing – review & editing. **Abdullah F. Aldairi:** Data curation, Formal analysis, Resources, Writing – review & editing. **Abdullah M. AlShahrani:** Writing – original draft, Writing – review & editing. **Abdullah F. Alghannam:** Data curation, Writing – original draft, Writing – review & editing. **Rwaa H. Abdulal:** Data curation, Methodology, Writing – original draft. **Abed H. Al-Slaihat:** Data curation, Formal analysis, Writing – original draft. **Maysoun S. Qutob:** Formal analysis, Investigation, Writing – original draft, Writing – review & editing. **Mahmoud E. Elrggal:** Conceptualization, Data curation, Writing – review & editing. **Mazen M. Ghaith:** Resources, Software, Supervision, Validation, Visualization, Writing – original draft, Writing – review & editing. **Firas S. Azzeh:** Conceptualization, Project administration, Writing – original draft, Writing – review & editing.

## Declaration of competing interest

The authors declare that they have no known competing financial interests or personal relationships that could have appeared to influence the work reported in this paper.
